# Identification and Localization of the First Known Proteins of the *Trypanosoma cruzi* Cytostome Cytopharynx Endocytic Complex

**DOI:** 10.3389/fcimb.2019.00445

**Published:** 2020-01-17

**Authors:** Nathan Michael Chasen, Isabelle Coppens, Ronald Drew Etheridge

**Affiliations:** ^1^Department of Cellular Biology, Center for Tropical and Emerging Global Diseases (CTEGD), University of Georgia, Athens, GA, United States; ^2^Bloomberg School of Public Health, Johns Hopkins University, Baltimore, MD, United States

**Keywords:** cytostome, cytopharynx, *Trypanosoma cruzi*, kinetoplastid, endocytic, endocytosis, pre-oral ridge, SPC

## Abstract

The etiological agent of Chagas disease, *Trypanosoma cruzi*, is an obligate intracellular parasite that infects an estimated 7 million people in the Americas, with an at-risk population of 70 million. Despite its recognition as the highest impact parasitic infection of the Americas, Chagas disease continues to receive insufficient attention and resources in order to be effectively combatted. Unlike the other parasitic trypanosomatids that infect humans (*Trypanosoma brucei* and *Leishmania* spp.), *T. cruzi* retains an ancestral mode of phagotrophic feeding via an endocytic organelle known as the cytostome-cytopharynx complex (SPC). How this tubular invagination of the plasma membrane functions to bring in nutrients is poorly understood at a mechanistic level, partially due to a lack of knowledge of the protein machinery specifically targeted to this structure. Using a combination of CRISPR/Cas9 mediated endogenous tagging, fluorescently labeled overexpression constructs and endocytic assays, we have identified the first known SPC targeted protein (CP1). The CP1 labeled structure co-localizes with endocytosed protein and undergoes disassembly in infectious forms and reconstitution in replicative forms. Additionally, through the use of immunoprecipitation and mass spectrometry techniques, we have identified two additional CP1-associated proteins (CP2 and CP3) that also target to this endocytic organelle. Our localization studies using fluorescently tagged proteins and surface lectin staining have also allowed us, for the first time, to specifically define the location of the intriguing pre-oral ridge (POR) surface prominence at the SPC entrance through the use of super-resolution light microscopy. This work is a first glimpse into the proteome of the SPC and provides the tools for further characterization of this enigmatic endocytic organelle. A better understanding of how this deadly pathogen acquires nutrients from its host will potentially direct us toward new therapeutic targets to combat infection.

## Introduction

The causal agent of Chagas disease, *Trypanosoma cruzi*, is an obligate intracellular parasite of the kinetoplastid family that infects upwards of 7 million people in the Americas with ~30% developing life-threatening clinical disease (WHO, 2015; Perez-Molina and Molina, 2018). Like many trypanosomatids that cause disease in humans, *T. cruzi* is characterized by having a dixenous (two-host) life cycle that alternates between the hematophagous triatomine insect vector and its endothermic vertebrate reservoir that includes humans. Although the acute stage of infection is generally controlled by a highly effective immune response, total clearance does not occur, resulting in a life-long and often debilitating chronic infection (Groom et al., 2017). We currently lack the basic tools to effectively combat this pathogen, as methods of diagnosis are unreliable and drug treatments (Nifurtimox and Benznidazole) are both highly toxic and unable to eliminate the infection entirely (Camandaroba et al., 2003; Mejia et al., 2012; Molina-Garza et al., 2014; Maguire, 2015; Kansiime et al., 2018). As with any attempt to control an infectious disease, a better understanding of *T. cruzi's* basic biology is necessary for the effective identification of the areas where these parasites are most susceptible to therapeutic intervention (Alvarez et al., 2016).

One of the most poorly understood aspects of *T. cruzi* biology centers around the question of how this parasite exploits host resources in order to proliferate. Some important clues, however, have come from phylogenetic analyses tracing the evolution of kinetoplastids and their transition from bacterivorous predators to obligatory parasites. Although the most heavily studied kinetoplastids are the disease causing parasites of humans and domesticated animals (*Trypanosoma* spp. and *Leishmania* spp.), these organisms originally evolved from environmentally ubiquitous free-living, flagellated protozoans of the Excavata supergroup (phylum Euglenozoa) (Simpson et al., 2002; Lukes et al., 2014). These early branching eukaryotes comprise a diverse family of flagellates characterized primarily by the presence of an “excavated” feeding groove used to funnel food into the mouth of the cell, a plasma membrane pore known as the cytostome. This pore is continuous with a single tubular invagination (cytopharynx) adjacent to the flagellar pocket that extends to the posterior end of the cell. Extracellular material captured via this feeding apparatus [referred to collectively here as the cytostome-cytopharynx complex (SPC)] is subsequently endocytosed and targeted for lysosomal degradation (Eger and Soares, 2012). The extant free-living relatives of trypanosomatids, broadly classified as bodonids (e.g., *Bodo saltans*), can be found in virtually any marine or freshwater habitat where they primarily filter feed using the SPC endocytic pathway to phagocytose their bacterial prey. It has therefore been assumed that SPC mediated phagotrophy of prokaryotes was, most likely, the ancestral mode of nutrient acquisition in the first kinetoplastids (Stevens, 2014; Flegontova et al., 2018). With the recent identification of the earliest branching monoxenous trypanosomatid, *Paratrypanosoma confusum*, as the “missing link” between *B. saltans* and the dixenous trypanosomatids (Simpson et al., 2002; Stevens, 2008), it has become clear that obligatory parasitism first began as an association with the arthropod lineage (propagated via fecal/oral transmission) and that the dixenous parasitism we see in the pathogenic trypanosomatids likely arose independently on several occasions. It is notable that the dixenous stercorarian trypanosomes (which includes *T. cruzi*) still rely on fecal transmission from the insect to their vertebrate host. This initial transition of free-living kinetoplastids into arthropod parasites coincided with an acute reduction in the size of their genome (~50% gene loss), which included the loss of many metabolic pathways (Opperdoes et al., 2016). This streamlining was also accompanied by the abandonment of their second flagellum (Deschamps et al., 2011; Harmer et al., 2018). Despite all these changes however, the SPC structure of monoxenous trypanosomatids has remained intact, suggesting a continued role in nutrient uptake suited for life in the insect gut. Curiously, in the second major transition of the pathogenic trypanosomatids to a dixenous lifestyle, *T. cruzi* alone retained the SPC whereas the salivarian lineages (*Trypanosoma brucei* and *Leishmania* spp.) abandoned this endocytic structure entirely (Porto-Carreiro et al., 2000; Field and Carrington, 2009).

Although we do not know definitively why *T. cruzi* has retained the SPC, there is clearly an interesting correlation between the distinct replicative niches inhabited by a parasite and its mode of nutrient uptake. *T. brucei*, for example, divides extracellularly in the bloodstream of mammals where the host provides simple amino acids and sugars at high levels that can be readily transported across its plasma membrane to facilitate growth (Mazet et al., 2013; Creek et al., 2015; Mathieu et al., 2017; Marchese et al., 2018). Apparently, only host iron sequestering proteins, such as transferrin, still need to be brought into the parasite cell directly via receptor-mediated endocytosis at the flagellar pocket (Coppens et al., 1987; Schell et al., 1991; Mach et al., 2013). *Leishmania* spp. on the other hand, do replicate within host cells, but rather than live freely in the cytoplasm, they reside within lysosome-like vacuoles of specialized phagocytic cells. As a result, *Leishmania* spp. occupy a low pH environment replete with proteases that actively digest host macromolecules into a milieu of simple nutrients (Alexander, 1975; Antoine et al., 1990; Russell et al., 1992). *T. cruzi* is therefore the only trypanosomatid that lives directly in the host cytoplasm and is one of the few protozoan parasites that inhabit the cytosol of nucleated cells, making this replicative niche a surprisingly rare choice (de Souza et al., 2010; Barrias et al., 2013). One possible reason for this rarity comes from recent work demonstrating that the cytosolic environment of host cells is far less conducive to pathogen growth than previously thought (Goetz et al., 2001). The cytosol, despite having abundant macromolecules, is deficient in freely available amino acids, when compared to the mammalian bloodstream or host cell lysosomes (O'Riordan and Portnoy, 2002; Abu Kwaik and Bumann, 2013). *T. cruzi* may have retained the SPC, in part, as a mechanism for amastigotes to harvest host cytoplasmic macromolecules as the fuel required for rapid parasite growth. Direct digestion of these materials via the SPC complex would provide *T. cruzi* with metabolites that are otherwise scarce in the host cytoplasm. This seemingly “non-specific” method of nutrient uptake could also explain how *T. cruzi* is able to grow and propagate in such a wide variety of hosts and cell types (Browne et al., 2017). Alternatively, the loss of the SPC in *T. brucei* or *Leishmania* sp. may be due to the fact that these organisms undergo critical developmental changes in the mid and foregut of their arthropod vectors whereas *T. cruzi* alone develops into its infectious stage in the hindgut as part of its stercorarian transmission lifestyle (reviewed in Goncalves et al., 2018). The SPC may therefore serve a critical role for trypanosomatids needing to successfully navigate the insect hindgut. It appears as though the fecal/oral transmission route for the monoxenous trypanosomatids like *P. confusum* is the ancestral form of transmission still retained by *T. cruzi* albeit as part of its vertebrate transmission strategy (Flegontov et al., 2013).

Our current understanding of the complex organization and dynamics of the SPC in *T. cruzi* is derived primarily from a combination of extensive electron microscopy studies including scanning, transmission and tomography based 3D reconstructions (Steinert and Novikoff, 1960; Milder and Deane, 1969; Preston, 1969; Martinez-Palomo et al., 1976; De Souza et al., 1978a,b; Souto-Padron and de Souza, 1983; Okuda et al., 1999; Vatarunakamura et al., 2005; Correa et al., 2007; Souza, 2009; Alcantara et al., 2014). First, the membrane tubule itself is embedded between two distinct sets of microtubule root fibers; one set of triplet microtubules initiating at the cytostome entrance and a second quartet of microtubules beginning at the centrosome near the base of the flagellar pocket, abutting the plasma membrane to form an intriguing surface prominence known as the pre-oral ridge (POR) before descending again into the cytosol and serving as a guide for the construction of the cell spanning SPC structure (Alcantara et al., 2014). In addition to its structural complexity, the SPC is also highly dynamic, as it disassembles in the infectious/non-dividing forms (metacyclics and trypomastigotes) and reconstitutes itself in the replicative stages (epimastigotes and amastigotes) (Vidal et al., 2016). It is also thought to break down during cytokinesis (Alcantara et al., 2017). Although the cytostome membrane pore and tubular invagination are lost during disassembly, the underlying microtubule structures appear more stable, potentially allowing for the rapid regeneration of this organelle (Alcantara et al., 2014). The ultimate result of these changes is that endocytosis ceases in the metacyclic and trypomastigote forms, only to become measurable again after stage conversion and cell division resumes (Vidal et al., 2016). Why the SPC disassembles in these non-replicative stages of *T. cruzi* has never been determined, but it may function as a defense against bringing in host antimicrobial proteins or more simply because the infectious forms are no longer replicating and have no need to consume additional complex metabolites. Despite the detailed structural analysis of the SPC, there is still a great deal that remains unknown about this primordial feeding apparatus including the identity of proteins responsible for its construction, function and capture of endocytosed cargo.

We report here the identification of the first known proteins specifically targeted to the enigmatic cytostome-cytopharynx complex in *T. cruzi*. Our initially identified SPC targeted protein (CP1), was endogenously Ty-epitope tagged and shown to localize with several fluorescently labeled cargo proteins, consistent with its targeting to the endocytic organelle. As expected, the CP1 localization to the SPC was dynamically regulated and disassembled in the infectious trypomastigote and reestablished in the replicative stages. Overexpressed CP1-mNeon-Ty underwent normal trafficking and thus facilitated visualization of the SPC for the first time in live parasites. When characterizing the localization of CP1, we observed that co-staining of the parasite surface with the mannose binding lectin Concanavalin A (ConA) allowed us to determine, using super-resolution light microscopy alone, the region of the SPC entrance and pre-oral ridge, which we suspect plays an instrumental role in the binding efficiency of nutrient endocytosis. Finally, our immunoprecipitation (IP) of CP1, followed by mass spectrometry (MS) analysis led to the identification of two additional SPC associated proteins (CP2 and CP3), which also label the protein endocytic pathway, thus validating their interaction with CP1. This work reveals a first glimpse into the proteome of the SPC and provides the necessary tools for further characterization of this unusual endocytic organelle.

## Materials and Methods

### Parasite Cultures

Epimastigotes of *T. cruzi* Y strain were cultured in LDNT/LIT medium (Kirchhoff et al., 1984) supplemented with 15% FBS. Aliquots of FBS (VWR, USDA certified) were freshly heat inactivated at 76°C for 40 min (to help improve epimastigote viability at low numbers) prior to addition to the media (this heat inactivated FBS will precipitate if frozen). To obtain amastigotes, 1 mL of maximum density epimastigotes were differentiated to metacyclic trypomastigotes via prolonged starvation in depleted LIT media (for at least 3 days). The resulting parasites were then incubated overnight with fresh FBS (with intact complement) to kill any remaining epimastigotes. The surviving metacyclic trypomastigotes were added to flasks containing human foreskin fibroblasts (HFF) grown in High Glucose Dulbecco's Modified Eagle's Medium (DMEM-HG) (Hyclone) supplemented with L-glutamine and 10% heat-inactivated (56°C) Cosmic Calf serum (CCS) (Hyclone). After 24–48 h media was replaced with medium containing only 1% (CCS) for further culturing.

### Generation of Cloning Constructs and Transfection

C-terminal tagging of CP1 was performed as described by Lander et al. (2016), the Ty epitope sequence, fused to a downstream hygromycin selection cassette, were first amplified with 40bp homology arms for insertion upstream of the CP1 stop codon (P1 and P2). This product was co-transfected with the SpCas9 vector generated by Lander et al. modified to contain a gRNA protospacer (P6) targeted to a site between the two chosen 40 bp homology regions. Mutants were selected with both Hygromycin (250 μg/mL) (Fisher Scientific) and G418 (400 μg/mL) (Corning). For overexpression of mNeon-Ty tagged proteins, we utilized the pTREX vector (Martinez-Calvillo et al., 1997) modified with a *T. cruzi* optimized and “fixed” Neomycin resistance cassette (IDT Geneblock) (see **Figure 3A**) that allowed us to stringently select with 2,000 μg/mL G418. We also modified the vector to contain a *T. cruzi* optimized mNeon-Ty sequence (Shaner et al., 2013) (IDT Geneblock). Final vector products were generated and assembled using standard Gibson assembly parameters (Gibson et al., 2009) and primers (CP1: P7,P8 CP2: P9,P10 CP3: P11,P12) using the NEB HiFi Assembly kit. P6 and P7 primers were utilized to amplify the vector for Gibson assembly. Parasites were transfected as described by Lander et al. (2015) using a BTX ECM830 (Harvard Apparatus). Primers used for cloning are identified in Supplementary Table 1.

### Western Blot Analysis and Immunofluorescence Assays

For Western blot lysates, 1 × 10^7^ epimastigotes were spun down at 1,200 × g then washed 1x in PBS. Pellets were then resuspended in 1x SDS PAGE sample buffer (LICOR) containing β-mercaptoethanol. Half of the sample (5 × 10^6^ epimastigote equivalent) was loaded into each well and Western blotting was performed via the general established protocol. Western blotting antibody dilutions: 1:500 for α-CP1 mouse and 1:1,500 for α-Ty mouse (mAb BB2) (Gay et al., 2016) LICOR 800 α-mouse was used as secondary antibody at 1:20,000 and images were taken in a BioRad Chemidoc. Total protein loading controls were imaged from stain-free TGX gels prior to membrane transfer. Immunofluorescence assays (IFA) were performed as previously described (Chasen et al., 2017) with modifications for epimastigotes. Epimastigote were washed in nutrient free pH 7.2 Triatomine Artificial Urine media (nfTAU) (Chiurillo et al., 2017) prior to fixation in nfTAU containing 3.5% paraformaldehyde (Electron Microscopy Sciences). Cold fixation solution was added to parasites and allowed to fix and equilibrate to room temperature for 20–30 min. Fixed parasites were allowed to adhere in nfTAU to poly-l-lysine coated cover slips for 30 min prior to proceeding with the IFA protocol. IFA antibody dilutions: 1:500 for α-CP1 mouse and 1:1,100 for α-Ty mouse monoclonal. α-mouse Alexafluor-488 and Alexafluor-568 antibodies were used as secondary antibodies. Coverslips were mounted to slides using Prolong Antifade mounting media (Invitrogen).

### Endocytosis Assays

For fluorescent epimastigote endocytosis assays, 1 × 10^7^ epimastigotes were chilled on ice and then spun down in a 4°C centrifuge at 1,200 g. Parasites were then resuspended in 500 μl of chilled complete TAU (pH 7.2) containing 25 μg/mL BSA-Rhodamine (Rockland Immunochemicals), 25 μg/mL Transferrin-488 (Molecular Probes), and 10 μg/mL α-Rabbit Alexafluor-568 IgG (Thermo Fisher) and then incubated at 28°C for 25 min. Parasites were then spun down at 4°C and the supernatant removed prior to resuspension of the pellet in 1.5 mL of 4°C nfTAU containing 4% paraformaldehyde (Electron Microscopy Sciences). Fixation was allowed to progress at room temperature for 30 min before spinning down for slide mounting and fluorescence imaging as described above.

For intracellular amastigote endocytosis assays, coverslips with HFF monolayers were treated with CellTrace CFSE as described in the commercial protocol (Invitrogen). After labeling, cells were washed three times and followed by a 3 h recovery in 10% FBS containing DMEM-HG. CP1-Ty trypomastigotes were then added to the culture and 48 hrs later amastigotes were lysed out of the cells using a 16-gauge needle. Amastigotes were fixed and attached to coverslips as described above for epimastigotes. Otherwise, IFA's were performed as described above using α-Ty primary antibody (mAb BB2) and Alexafluor-568 secondary (Thermo Fisher). For cytochalasin B experiments, epimastigotes were pre-treated for 10 min at 28°C with cytochalasin B (300 μM, Enzo Lifesciences) prior to performing the endocytosis assay. Cytochalasin B concentration was maintained during entirety of the assay.

### Concanavalin a Labeling of mNeon Overexpression Mutants

Prior to fixation, 1 × 10^7^ epimastigotes of the mNeon overexpression lines were chilled, spun at 1,200 g, and washed 1x ice-cold nfTAU (pH 7.2). They were then incubated on ice in TAU (pH 7.2) containing 10 μg/mL Rhodamine Conjugated Concanavalin A (ConA) (Vector Laboratories). After spinning down in a pre-chilled 4°C centrifuge, the supernatant was removed, and the pellet was immediately resuspended in 1.5 mL of cold fixative (4% paraformaldehyde in nfTAU pH 7.2) and allowed to equilibrate to room temperature for 30 min, fixed parasites were resuspended in nfTAU prior to adhering to coverslips as described above.

### Fluorescent Microscopy

IFA and routine fluorescence images were taken using the Zeiss Elyra S1 Structured Illumination Microscope in the Center for Tropical and Emerging Diseases microscopy core.

### Immunoelectron Microscopy

CP1-Ty epimastigotes were washed twice with PBS before fixation in 4% paraformaldehyde (Electron Microscopy Sciences, PA) in 0.25 M HEPES (pH 7.4) for 1 h at room temperature and then in 8% paraformaldehyde in the same buffer overnight at 4°C. Parasites were pelleted in 10% fish skin gelatin, and the gelatin-embedded pellets were infiltrated overnight with 2.3 M sucrose at 4°C and frozen in liquid nitrogen. Ultrathin cryosections were incubated in PBS and 1% fish skin gelatin containing mouse α-Ty antibody at a 1/100 dilution and then exposed to the secondary antibody that was revealed with 10-nm protein A-gold conjugates. Sections were observed and images were recorded with a Philips CM120 electron microscope (Eindhoven, the Netherlands) under 80 kV.

### Analysis of Sequence, Alignment, Tree Generation, and Structure Prediction

Alignments were generated using T-Coffee (Madeira et al., 2019) and figures were generated using Jalview 2 software (Waterhouse et al., 2009). Tree was generated in Geneious Prime using the Jukes-Cantor and neighbor-joining parameters with the *B. saltans* CP2 related sequence as an outgroup. Consensus tree was generated using a random seed and 100 bootstrapped replicates with a support threshold of 90%. Structure predictions were generated using the I-TASSER server (Yang and Zhang, 2015).

### Purification of Recombinant Antigen and Mouse Polyclonal Antibody Generation

The chosen antigenic region of CP1 (CP1Ag) was identified using the IEDB suite of antigenicity prediction software (http://tools.immuneepitope.org/bcell/) (Chou and Fasman, 1978; Emini et al., 1985; Karplus and Schulz, 1985; Parker et al., 1986; Kolaskar and Tongaonkar, 1990; Bogitsh et al., 1995; Larsen et al., 2006; Jespersen et al., 2017). The DNA sequence for CP1Ag was amplified from *T. cruzi* genomic DNA and cloned into the pET-32 LIC/EK vector (Novagen), which adds an N-terminal thioredoxin and histidine tag to the expressed protein. Recombinant CP1Ag was expressed and initially purified via a nickel-affinity column (HisPur Thermo Fisher) as previously described (Chasen et al., 2017). This initial purified fraction was digested using biotinylated thrombin to cleave the N-terminal thioredoxin-tag and histidine tag. After thrombin removal, the antigen was again passed through the nickel column and the purified tag-less antigen was gently eluted using 10 mM imidazole, leaving the tag attached to the column.

Antibodies in mice were generated as previously described (Chasen et al., 2019). Swiss Webster mice (Charles River) were inoculated intraperitoneally with 100 μg of Cp1Ag mixed with complete Freund's adjuvant, followed by two boosts with 50 μg of CP1-Ag, with each boost being mixed with incomplete Freund's adjuvant. The final serum was collected by cardiac puncture after CO_2_ euthanasia. The animal protocol used was approved by the UGA Institutional Animal Care and Use Committee (IACUC).

## Co-immunoprecipitation and Mass-Spectrometry

For each immunoprecipitation, a 50 mL culture of epimastigotes was grown to mid-log phase (1-2x10^7^ epimastigotes/mL) and a co-immunoprecipitation using the Ty peptide for elution was performed as described by Huet et al. (2018). For identification of excised bands, matrix assisted laser desorption ionization (MALDI) was performed using a Bruker Autoflex (TOF) mass spectrometer. Shotgun mass spectrometry of eluates was performed on an Orbitrap Elite system with ESI ion source and an interchangeable nanospray source.

## Epimastigote Growth Assays

2.5 × 10^6^ epimastigotes of each strain were resuspended in LIT containing 15% heat inactivated FBS. Counting was performed every 24 h using a Coulter Counter (Beckman Coulter). Growth assays were performed in triplicate and fold-change significance was determined via unpaired *t*-tests using the Prism Software Suite.

## Results

### Identification of the First Cytostomal Protein (CP1) Specifically Targeted to the Cytostome-Cytopharynx Endocytic Organelle

**Figure 1:**
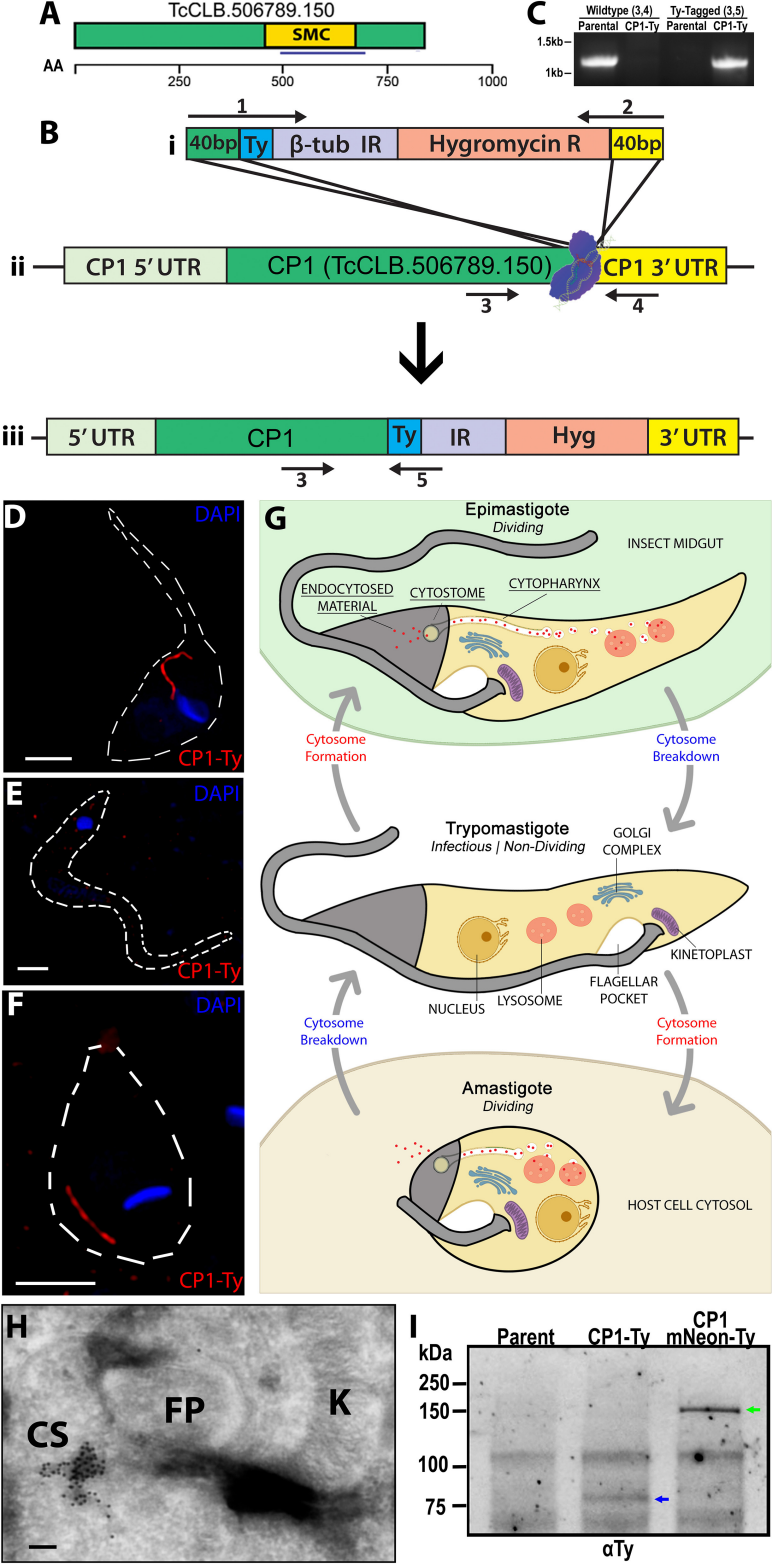
Endogenous Tagging of a cytostome-cytopharynx complex Protein. **(A)** Schematic of CP1 with annotated structural maintenance of chromosome domain (SMC) and chosen antigenic region for later antibody generation (blue underline) (see Supplementary Figure 5). An amino acid scale is below the schematic. **(B)** Cartoon showing CRISPR Cas9 C-terminal tagging strategy used to tag CP1. A guide RNA with a protospacer targeted to the CP1 3′ UTR near the ORF directs spCas9 to form a double stranded break **(B**ii**)**. A Ty-tagging repair template containing 40 bp homology, for both the 3′ end of the CP1 ORF and the 3′ side of the cut-site **(B**i**)**, was co-transfected to endogenously tag the CP1 locus **(B**iii**)**. **(C)** PCR of genomic DNA from CP1-Ty mutants confirms the C-terminal tagging of both endogenous CP1 loci. **(D–F)** SR-SIM IFA showing the linear structure labeled by CP1-Ty in an epimastigote **(D)** and amastigote **(F)**. In **(E)**, a trypomastigote lacking the CP1-Ty labeled structure is shown. **(G)** Cartoon showing the structure and breakdown of the SPC in the different *T*. cruzi life cycle stages. **(H)** Cryogenic immunoelectron microscopy of an epimastigote showing immuno-gold labeling of CP1-Ty at the cytostome (CS). Gold particles are 10 nm. **(I)** αTy immunoblot of lysates from Parental, CP1-Ty, and CP1-mNeon-Ty overexpression mutants shows a faint band of CP1-Ty (blue arrow) and a strong band of overexpressed CP1-mNeon-Ty (green arrow) Scale bars: IFA 2 μm, EM 100 nM.

As part of an ongoing epitope tagging and localization screen being conducted in the Y-strain of *T. cruzi*, we utilized a CRISPR/Cas9 mediated endogenous tagging strategy as described by Lander et al. whereby the Cas9 nuclease, in combination with a gRNA targeting the 3′ end of the hypothetical protein (TriTrypDB: TcCLB.506789.150, NCBI Accession: XP_819915.1) (Figure 1A) was transfected into parasites. This was followed by dual selection of both a repair template containing the introduction of a C-terminal Ty-epitope (Hygromycin) and the gRNA/Cas9 expression vector (G418) (Figure 1B). A diagnostic PCR to assess the loci specific modifications to the genomic DNA demonstrated a product of the predicted size for the epitope tagged locus only in the tagged strain with no products being observed for the parental locus, confirming the successful tagging of both genomic loci (Figure 1C). The annotated open reading frame (ORF) of this gene, which we have renamed CP1, encodes for an 841 amino acid (aa) protein, with a predicted isoelectric point of 5.57 and a predicted molecular weight of 93.0 kDa. Although annotated as a hypothetical protein, CP1 does contain a predicted structural maintenance of chromosome (SMC) domain (TIGR02168, 455-670 aa) (Figure 1A) most likely due to the presence of a potential coiled coil structure. This protein lacks any orthologs outside the closely related *Trypanosoma theileri, Trypomanosoma rangeli*, and *Trypanosoma grayii*. A western blot of CP1-Ty epimastigote lysate showed CP1-Ty to migrate at a size of ~80 kDa (Figure 1I), which is below the predicted molecular weight of 93 kDa, potentially indicating N-terminal processing or post-translation modification of CP1-Ty. In images from immunofluorescence assays (IFA) taken using super-resolution structured illumination microscopy (SR-SIM), we noted the localization of CP1-Ty to a distinct linear structure that was present in both of the replicating stages, the epimastigote and amastigote (Figures 1D,F), but was absent in the non-replicating trypomastigote stage (Figure 1E). This structure, and its stage-specific nature, were reminiscent of the endocytic organelle of *T. cruzi*, referred to here as the cytostome/cytopharynx complex (SPC) (summarized in Figure 1G). Cryogenic immuno-electron microscopy (CryoIEM) analysis of CP1-Ty also showed its localization to convoluted, multibranched tubular structures in proximity to the flagellar pocket (FP) and kinetoplast (K) (Figure 1H).

### CP1 Labels the Protein Endocytic Pathway of the Cytostome-Cytopharynx Complex

**Figure 2:**
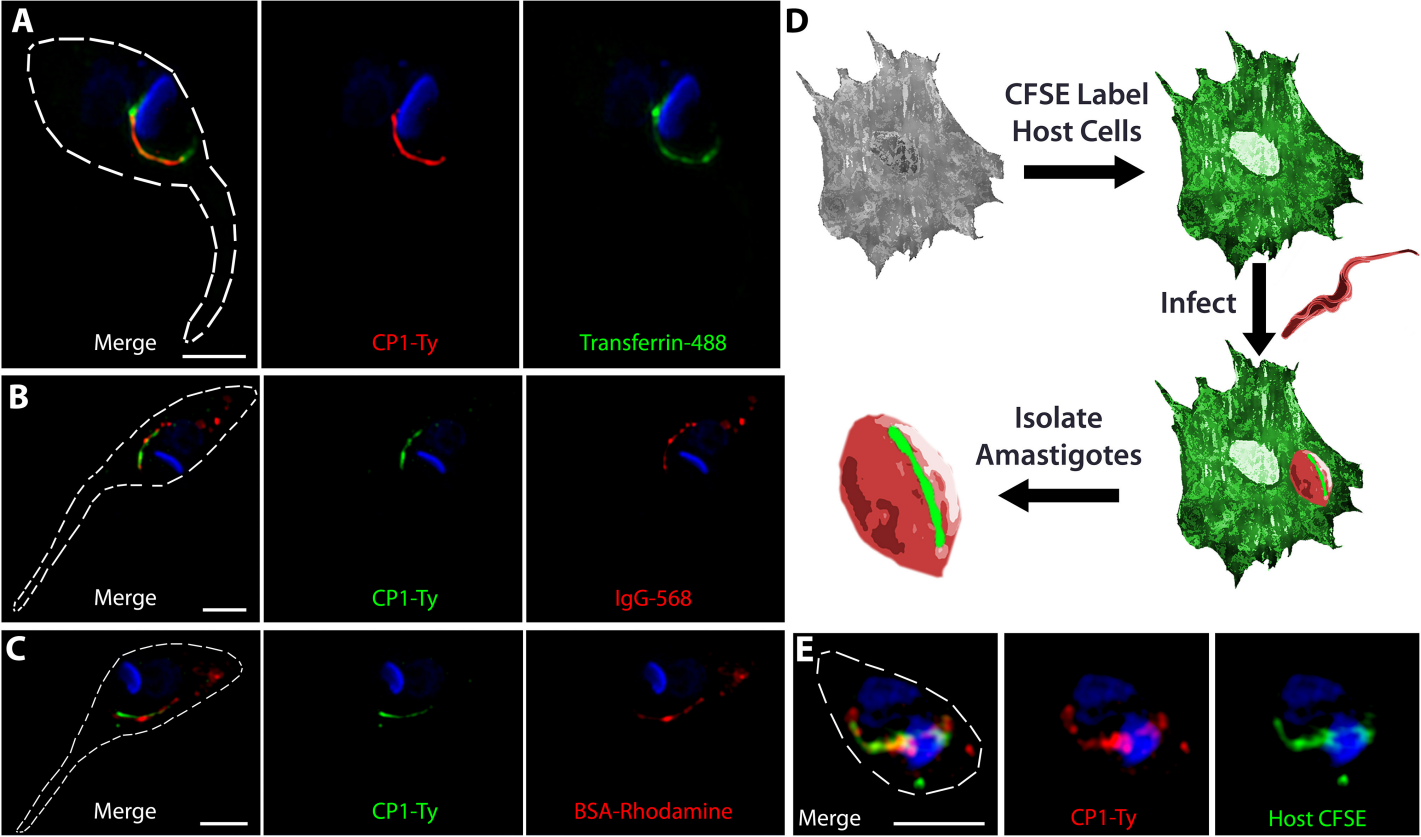
CP1 labels the protein endocytic pathway of the cytostome/cytopharynx complex. SR-SIM IFAs of epimastigotes after endocytosis of Transferrin (**A**, green), IgG (**B**, red), and BSA (**C**, red), shows labeling of the SPC endocytic structure by CP1-Ty. **(D)** Strategy for evaluating amastigote endocytosis of host-cell cytosolic protein labeled by CFSE. **(E)** SR-SIM IFA of an amastigote that has endocytosed CFSE labeled host cytosolic protein, coinciding with the SPC labeled by CP1-Ty. Scale bars: 2 μm.

As previous work in the field supported the role of the SPC as the primary route of protein endocytosis in *T. cruzi* epimastigotes, we performed endocytosis assays to confirm that the CP1 labeled structure was targeting to this endocytic organelle. We fed epimastigotes fluorescent Transferrin (Figure 2A), goat Immunoglobin (IgG) (Figure 2B), and Bovine Serum Albumin (BSA) (Figure 2C) to label the endocytic pathway and observed, through co-staining, that CP1-Ty labeled the endocytic pathway of the SPC for all three proteins. The SPC is remarkable in its apparent lack of specificity for cargo as it appears capable of endocytosing any protein we provide. It is notable that we only observed endocytosis via the SPC and failed to detect protein uptake through the flagellar pocket. This is in stark contrast to *T. brucei* which endocytoses exclusively through its flagellar pocket and to previously published reports of *T. cruzi* (de Figueiredo and Soares, 2000; Kalb et al., 2014). In order to evaluate endocytosis in intracellular amastigotes, we stained host cells first with the free-amine labeling fluorescent dye CFSE followed by infection with trypomastigotes from the CP1-Ty tagged line. After conversion to replicative amastigotes and growth for 48 h, amastigotes were released mechanically, fixed and stained with α-Ty antibodies (summarized in Figure 2D). We observed that endocytosed CFSE labeled host-cell protein colocalized with the CP1-Ty labeled structure in amastigotes, further confirming that this structure was the endocytic organelle (Figure 2E). These images also showed, for the first time, that amastigotes endocytose human host-cell cytosolic protein via the SPC.

### Fluorescently Tagged and Overexpressed CP1 Traffics to the Cytostome-Cytopharynx Complex

**Figure 3:**
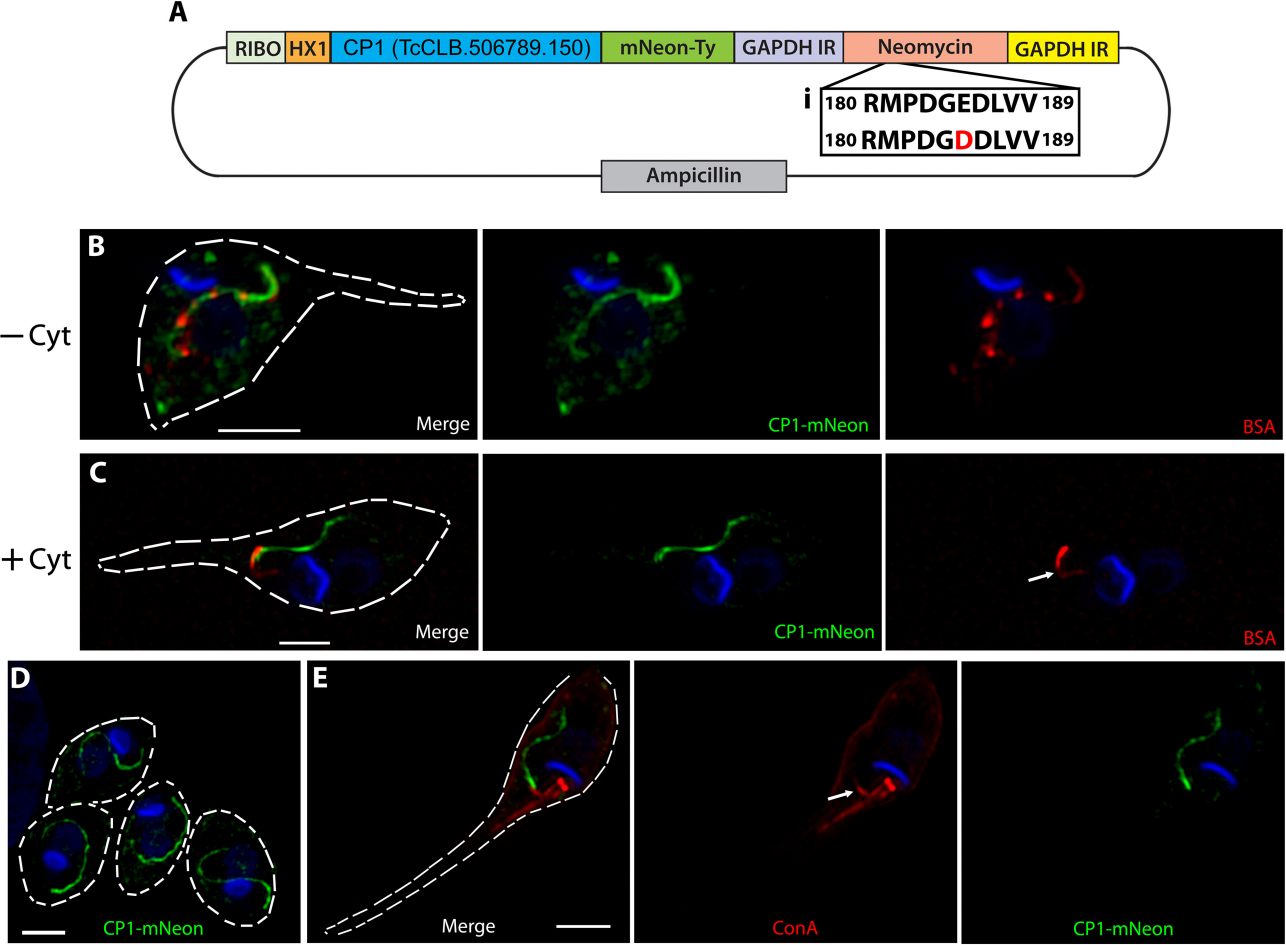
Overexpressed CP1-mNeon-Ty Localizes to the SPC. **(A)** Vector map of the CP1-pTREX overexpression plasmid with a common detrimental mutation of the neomycin cassette restored (i). This fixed neomycin cassette allows for harsh G418 selection of epimastigotes at up to 2,000 μg/mL. **(B)** SR-SIM microscopy of a CP1-mNeon epimastigote after the endocytosis of BSA-Rhodamine shows that BSA and CP1-mNeon label the same endocytic pathway. **(C)** Treating epimastigotes with cytochalasin B during the assay prevented endocytosis via the SPC, causing BSA to strongly label the pre-oral ridge in front of the cytostome (arrow). **(D)** SR-SIM of amastigotes expressing CP1-mNeon-Ty shows SPC labeling. **(E)** SR-SIM images of epimastigotes expressing CP1-mNeon-Ty, showing SPC labeling. Labeling at 4°C with Concanavalin A - Rhodamine was used to identify the entrance to the SPC (arrow). Scale bars: 2 μm.

When cloning the CP1-mNeon-Ty overexpression constructs (Figure 3A) we sequenced the drug marker of the pTREX vector and observed a G → T point mutation in the conserved catalytic region of the neomycin phosphate transferase II protein (NPTII). This mutation results in a substitution of an aspartic acid for the glutamic acid at position 182 and is known to dramatically reduce the resistance conferred by NPTII (Yenofsky et al., 1990). It had been suggested in this prior report that this less active form of the neomycin drug resistance marker may have been inadvertently propagated throughout numerous constructs in multiple research communities. We hypothesized that this mutation may be the cause for the lackluster G418 resistance conferred to epimastigotes (200–400 μg/mL) (Torres-Silva et al., 2018), which results in slow killing of wild type parasites lacking the cassette as well as potentially forcing the massive duplication and ultimately exaggerated overexpression by this vector in the resulting stable transfectants. To decrease the time of selection after transfection, we replaced the NPTII coding region with a *T. cruzi* codon-optimized version lacking the E182D substitution and increased drug selection pressure (Figure 3A, inset). This “fixed” version of NPTII allowed selection of our CP1-mNeon-Ty overexpressing epimastigotes with higher drug selection conditions up to 2,000 μg/mL of G418. Western blot analysis of lysate from this mutant population showed a strong band around the predicted molecular weight of 132 kDa (Figure 1I). SR-SIM microscopy revealed that the fusion protein of CP1-mNeon-Ty trafficked normally in epimastigotes as it colocalized with endocytosed BSA-Rhodamine along the length of the endocytic pathway (Figure 3B) When these mutants were pre-treated with Cytochalasin B, we observed that endocytosis was prevented (Figure 3C) as has previously been described (Bogitsh et al., 1995). As observed in this endocytosis blocking experiment (+Cyt), BSA clearly binds strongly to a region adjacent to the SPC entrance, also known as the pre-oral ridge (POR), which lies between the entrance of the flagellar pocket and the cytostome. The binding of a number of proteins at the POR prior to being endocytosed has been observed previously in primarily EM experiments (Vidal et al., 2016). It was also observed in our localization experiments, that in the absence of a permeabilization step, the endocytosed cargo displayed a markedly packet-like appearance (Figure 3B, see BSA-Rhodamine) as opposed to the continuous linear presentation of cargo observed when cells are permeabilized (see Figure 2A). When amastigotes expressing CP1-mNeon-Ty were imaged within host cells, the same tubular pattern reminiscent of the SPC was again observed (Figure 3D). By combining the CP1-mNeon-Ty expressing parasites with Concanavalin A (ConA) staining at 4°C, followed by fixation, we observed a unique labeling of two linear structures emerging from the flagellar pocket with one following the parasite body along the flagellar attachment zone and the other stopping abruptly at the POR and SPC entrance. This staining pattern seemed to confirm for the first time using light microscopy, what had been seen in prior reports using EM techniques, that the POR contains a concentration of mannose containing glycans (Figure 3E arrow) (Martinez-Palomo et al., 1976). The CP1-mNeon-Ty overexpressing parasites also allowed us to validate previous findings that parasites undergoing division (Supplementary Figure 1A) and infectious trypomastigotes (Supplementary Figure 1B) lack defined SPC structures (Alcantara et al., 2017).

### Identification of Two Additional Cytostome-Cytopharynx Proteins Associated With CP1

**Figure 4:**
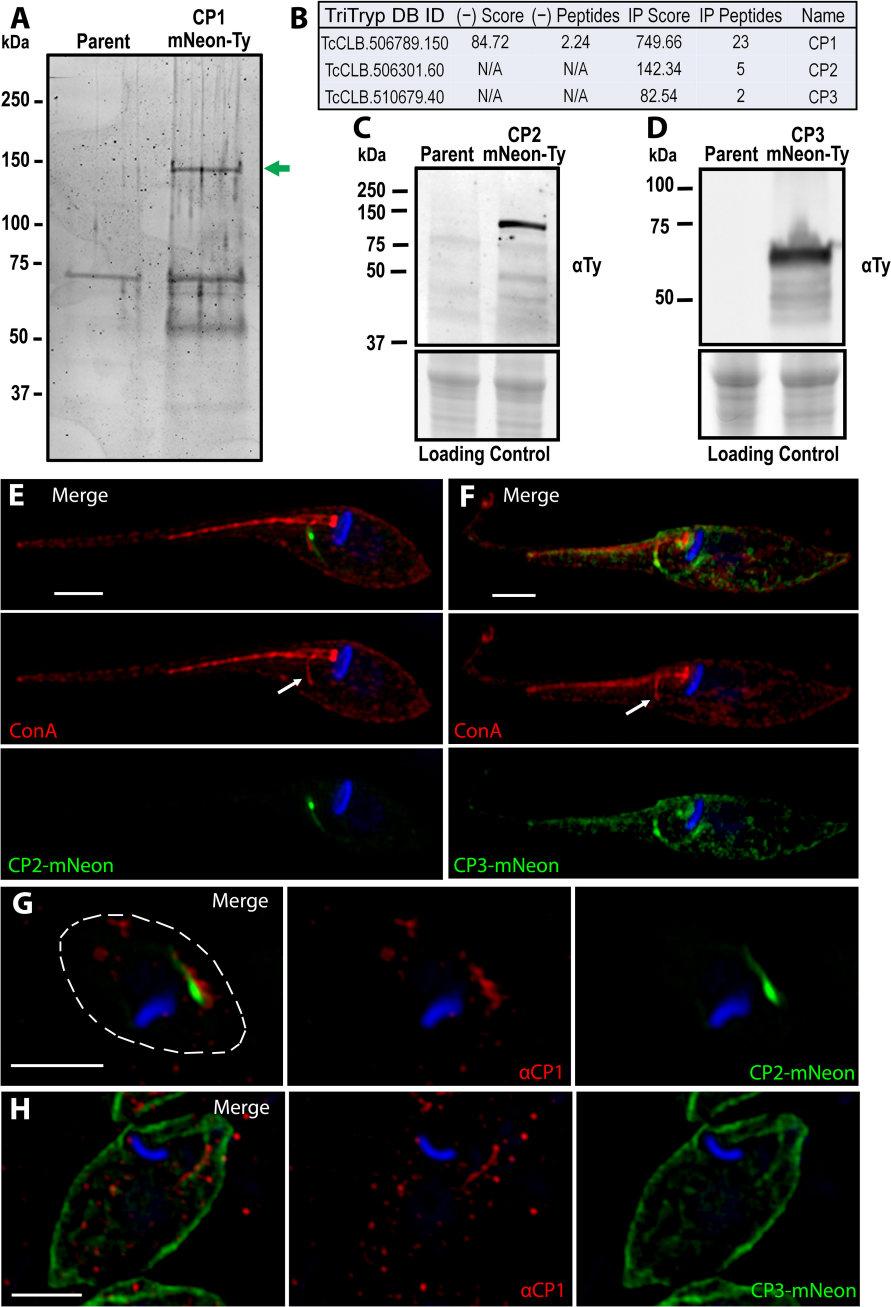
Identification of the CP1 associated proteins CP2 and CP3. **(A)** SDS page gel of CP1-mNeon-Ty CoIP eluate stained for total protein. Several bands are present in the CoIP that are absent from the control IP using parental lysate. Band excision and MALDI mass-spectroscopy (MS) analysis confirmed that the upper band (green arrow) is CP1-mNeon-Ty. **(B)** Notable hits from Orbitrap shotgun MS of the CP1 CoIP eluate revealed two hypothetical protein (CP2, CP3) in addition to CP1. αTy immunoblots of lysates from CP2-mNeon-Ty **(C)** and CP3-mNeon-Ty **(D)** overexpressing epimastigotes show that the tagged protein are expressed. **(E,F)** SR-SIM of CP2-mNeon-Ty and CP3-mNeon-Ty overexpressing mutants showing SPC labeling. 4°C labeling of with Concanavalin A -Rhodamine labels the SPC pre-oral ridge (white arrows). CP2-mNeon-Ty **(G)** and CP3-mNeon-Ty **(H)** in amastigotes also localize to the SPC, labeled by αCP1. Scale bars: 2 μm.

Taking advantage of our identification of CP1, we performed an immunoprecipitation to identify other SPC proteins for the continued characterization of this organelle and its structural components. We generated soluble lysate under native preserving conditions from our CP1-mNeon-Ty overexpressing parasite line and performed a pulldown using α-Ty antibody conjugated to paramagnetic beads with protein complexes being eluted through the use of excess Ty peptide. Eluates were run on an SDS-PAGE gel and proteins were visualized using BioRad Oriole Total Protein Stain. Individual band analysis combined with MALDI based-mass spectrometry demonstrated that the uppermost band was CP1-mNeon-Ty, confirming a successful pulldown (Figure 4A). Whole complex liquid chromatography-tandem mass spectrometry (LC-MS/MS) analysis revealed two additional proteins of interest that were both absent from the control and with a notable lack of orthologs in the SPC-less *T. brucei* and *Leishmania* spp. (Figure 4B).

The first target to be characterized, CP2 (TcCLB.506301.60 NCBI Accession: XP_807919.1), is a hypothetical protein that, like CP1 is found only in the closely related *Trypanosoma* species, as well as within the earliest branching trypanosomatid *P. confusum*, which retains the SPC (Skalicky et al., 2017). This protein is 720 aa long with a predicted molecular weight of 80 kDa and isoelectric point of 5.02. CP2, like CP1, also contains a predicted SMC domain (TIGR02168, 35-307 aa) (Supplementary Figure 2A). The C-terminal region of CP2 is also highly conserved amongst all predicted orthologs (675–720 aa) (Supplementary Figure 2B). This region has an I-TASSER predicted structure (Yang and Zhang, 2015) similar to that of a coiled-coil domain (Supplementary Figure 2C) which suggests the region may play a role in protein-protein interactions or oligomerization (Truebestein and Leonard, 2016). Notably, a 14-residue region of this domain (700–713 aa) (Supplementary Figure 2B, underline) is perfectly conserved amongst the predicted CP2 orthologs, even in the more distantly related *Paratrypansoma confusum* (Supplementary Figures 2E, 3). This suggests that the region plays a significant role in the protein's function or targeting, as it is under significant selective pressure to resist even minor modifications. To test the potential role of this 45 aa conserved region of CP2 in SPC targeting, it was fused to the C-terminus of mNeon (mNeon-CP2ct) and transiently overexpressed in epimastigotes. We observed, via fluorescence microscopy, an SPC-like structure resulting in transfected parasites, suggesting a role for this region in targeting CP2 to the cytostome (Supplementary Figure 2D). The second protein of interest, CP3 (TcCLB.510679.40, NCBI Accession: XP_807127.1) exists as multiple copies in several *T. cruzi* strains (i.e. Sylvio, Dm28C, TCC) but was found to exist only as a single copy in the CL Brener strain. Orthologs of CP3 were present only in the closely related species to *T. cruzi*, as was observed with CP1. CP3 is predicted to be 455 aa in length with a molecular weight of 52 kDa and isoelectric point of 8.96. Unlike in CP1 and CP2, no identifiable domains were found within this protein sequence.

We generated mutants overexpressing CP2-mNeon-Ty and CP3-mNeon-Ty and immunoblots revealed bands of ~110 kDa (Figure 4C) and 60 kDa (Figure 4D) respectively. The CP2 band matches the predicted molecular weight for CP2-mNeon-Ty (108 kDa), but the CP3 band is smaller than the 80 kDa predicted molecular weight, suggestive of post-translational processing that may affect its migration (Figure 4E). In CP2-mNeon-Ty expressing epimastigotes, this protein presents a strong signal near the SPC entrance as well as along the tubule itself (Figure 4E). CP3-mNeon-Ty also localized to the SPC in epimastigotes, but additionally localized diffusely to disparate areas of the cell which may be due to overexpression (Figure 4F). The dispersed labeling of CP3 suggests a potential minor affinity for cytoskeletal microtubules when overexpressed (Angelopoulos, 1970) however because the most intense labeling is along the SPC it suggests that CP3 may be labeling one or both of the microtubule root fibers associated with the endocytic structure that can also be seen in Supplementary Figure 4 (Kalb et al., 2014; Alcantara et al., 2017). To demonstrate the co-localization of CP2 and CP3 fusions to the SPC in amastigotes, we generated a polyclonal mouse antibody against a 240 amino acid antigenic region of CP1 recombinantly expressed in *E. coli* (Supplementary Figure 5A) (α-CP1). This region was identified through the combined use of the 7 IEDB antibody epitope prediction analysis methods (see Materials and Methods section). This region was further confirmed to not contain epitopes that had significant sequence similarity to other proteins in human cells or *T. cruzi*. This antigen (CP1Ag) was purified and isolated from the N-terminal tag prior to mouse inoculation (Supplementary Figure 5B). Immunoblots with α-CP1 recognized CP1-mNeon-Ty in lysates (Supplementary Figure 5C). The antibody was also capable of labeling endogenous levels of CP1 in IFAs of wildtype epimastigotes (Supplementary Figure 5D). Labeling of the SPC structure with α-CP1 demonstrated a clear overlap with both CP2 and CP3-mNeon-Ty in intracellular amastigotes (Figures 4G,H). We observed a mild but significant difference in the growth rates of CP3-mNeon-Ty overexpressing epimastigotes relative to the parental line (Supplementary Figure 6).

## Discussion

Here we report the identification and initial characterization of the first three known molecular components of the cytostome/cytopharynx complex (SPC) in *T. cruzi*. Given that the SPC is responsible for the endocytosis of extracellular material during the replicative epimastigote and amastigote stages, it is hypothesized to be a critical organelle for obtaining the necessary nutrients to support the high energetic demands of parasite replication. Our initial identification of CP1 was made possible by the recent development of efficient genomic modification tools (e.g., CRISPR/Cas9) in this parasite that we have employed in an ongoing protein localization screen. Our examination of endogenously tagged CP1-Ty showed its targeting to a linear structure seen only in the replicating forms and absent from the infectious stages, in line with previous examinations of the parasite endocytic structure (Vidal et al., 2016). In fluorescence-based protein feeding assays, CP1 co-localized with a variety of endocytosed cargo, confirming it to be a bona fide SPC resident protein. These studies highlighted the broad specificity of the SPC by showing that it was capable of shuttling BSA, IgG, Transferrin, and CFSE labeled host protein through this endocytic pathway. Surprisingly, we failed to observe any endocytosis of cargo, including albumin (BSA), via the flagellar pocket in our assays suggesting that the SPC may be the exclusive endocytic route for whole protein uptake in this parasite (Soares and de Souza, 1991; de Figueiredo and Soares, 2000; Kalb et al., 2014).

The overexpression of a CP1-mNeon-Ty fusion demonstrated that neither targeting nor viability was compromised as a result of the large fluorescent tag. This fusion product facilitated the first analysis of the SPC under native conditions and showed that the near-linear stream of endocytosed cargo seen in our initial feeding assays may be, in part, an artifact of sample permeabilization as parasites free from this processing step contained a regular array of repeating vesicle-like structures along the CP1 labeled SPC organelle. This also suggested the presence of two distinct functional subregions in the structure labeled by CP1: a short funnel-shaped cavity at the entrance followed by an array of regularly spaced budded vesicles being trafficked to the lysosomes for digestion. Additionally, while images showing pre-oral ridge (POR) labeling by Concanavalin A has been shown in electron microscopy images (Martinez-Palomo et al., 1976; De Souza et al., 1978b), this is the first demonstration of its use to label the POR in fluorescence light microscopy and this method can now be used to dissect this surface region's unique properties in future studies. In this and prior work, the POR often presents as a “sticky” patch on the cell surface where endocytosed cargo, even in the presence of endocytic blocking agents (e.g., CytB), binds specifically. Intriguingly, this POR region may function in a manner analogous to many free-living bacterivorous protozoa that coat the area around their cytostome entrance with lectins in order to enhance the capture of bacterial prey (Roberts et al., 2006; Wootton et al., 2007; Martel, 2009). It is tempting to speculate that, in addition to the organelle itself, *T. cruzi* may have retained surface receptor-like molecules to capture protein cargo and thus increase the efficiency of endocytosis like its predatory ancestors. Transmission EM analyses have also shown that the POR ridge surface membrane composition is distinctly lacking in transmembrane proteins yet is extremely glycan rich in comparison to the rest of the parasite membrane and appears to originate from the flagellar pocket itself (Martinez-Palomo et al., 1976). The river-like appearance of the POR membrane connecting the cytostome to the flagellar pocket suggests a potential mechanism to preserve overall membrane homeostasis whereby the membrane being “pulled” into the SPC originates from vesicle fusion events within the flagellar pocket itself (Souto-Padron and de Souza, 1983). A continued dissection of the role of the POR membrane and its associated surface proteins may shed light on how the process of SPC mediated endocytosis functions and is regulated.

In this work we also report the initial characterization of SPC targeted proteins derived from IP and MS analyses. Associated with CP1, we identified two additional hypothetical proteins named CP2 and CP3 that target to the SPC as shown through co-localization with our in-house derived α-CP1 antibody. While the hypothetical nature of CP1, CP2, and CP3, make it more challenging to predict the role they play in endocytosis, future studies will involve the generation of either deletion or conditional knockdown mutants to elucidate their function. We suspect that CP2 may be the most important of the proteins for SPC function, due to the presence of an ortholog in its more distant relative, *P. confusum*. CP2 also presents a highly conserved C-terminal region of 14 perfectly conserved amino acids. This region may serve as either a trafficking signal or an important interacting domain that does not tolerate amino acid changes despite potentially hundreds of millions of years of divergent evolution. (Marks et al., 1995; Nishimura and Balch, 1997; van Hennik et al., 2003; Bedoukian et al., 2008; De Marcos Lousa et al., 2012; Starodubova et al., 2017). The C-terminal region's predicted similarity to a coiled-coil domain also lends credence to it serving a role in protein-protein interactions (Truebestein and Leonard, 2016). Continued mutagenesis and fusion protein studies with this domain will help uncover the functional significance of this conserved region.

It is reasonable to expect that the SPC is composed of a variety of additional proteins that contribute to both its form and function and we believe that future in-depth characterizations of these proteins will increase our understanding of the *T. cruzi* endocytic machinery and its role in the parasite life cycle and pathogenesis. With this being the first report of proteins specifically targeted to the SPC structure in this or any organism, we now have the necessary tools to initiate an examination of the mechanistic underpinnings of this unique protozoan organelle.

## Data Availability Statement

All datasets generated for this study are included in the article/Supplementary Material.

## Author Contributions

NC and RE designed and performed the experiments, analyzed the data, and generated the figures. IC performed immuno-electron microscopy experiments and analysis. RE and NC wrote the manuscript with author input.

### Conflict of Interest

The authors declare that the research was conducted in the absence of any commercial or financial relationships that could be construed as a potential conflict of interest.

## Supplementary Material

Supplementary Figure 1 SPC labeling is absent during cytokinesis and the trypomastigote stage. **(A)** Amastigote labeling of the SPC by CP1-mNeon (ii) is absent in amastigotes undergoing cytokinesis (i), determined by the presence of a cleavage furrow (red arrow) and two nuclei (asterisk). **(B)** The SPC labeling by CP1-mNeon seen in amastigotes (ii), is absent in a trypomastigote (i). Scale bars: 2 μm.

Supplementary Figure 2 The highly conserved C-terminal region of CP2 is sufficient to traffic mneon to the cytostome/cytopharynx complex. **(A)** Cartoon showing CP2 and its predicted SMC domain over an amino acid scale line. The red line annotates the highly conserved C-terminal region (675–720 aa). **(B)** Alignment of the highly conserved C-terminal region. The black line identifies a notable 14 amino acid region that maintains 100% identity among all orthologs, even in the more distantly related *P. confusum*. **(C)** The ITASSER predicted structure of the conserved C-terminal region is similar to that of a coil-coil protein interaction domain. The confidence score (range −5.0 lowest to +1.0 highest) of the predicted structure is high at −0.7. **(D)** Fluorescence image of an epimastigote overexpressing mNeon fused to the 45 aa CP2 C-terminal region (see **B**) shows SPC labeling (arrow). **(E)** A bootstrapped Jukes-Cantor neighbor-joining tree analysis of CP2 orthologs using the alignment from Supplementary Figure 3. The weakly related potential ortholog from *B*. *saltans*, which is missing the C-terminal region entirely was used as the outgroup. Branches are labeled with their bootstrap value.

Supplementary Figure 3 Alignment of CP2 orthologs for tree analysis. T-coffee alignment of the CP2 orthologs from *T. cruzi CL Brener* (NCBI: XP_807919.1), *T. cruzi Dm28c* (Genbank: ESS69597.1)*, T.cruzi Sylvio* (Genbank: EKG03371.1)*, T. cruzi Marinkellei* (Genbank: EKF31948.1)*, T. rangelli* (Genbank: ESL08911.1)*, T. Theileri* (NCBI: XP_028885643.1)*, P. confusum* (TriTrypDB: PCON_0003310)*, B. saltans* (Genbank: CUG86305.1).

Supplementary Figure 4 CP3-mNeon labels structures reminiscent of the microtubule root fibers. Fluorescent image of the CP3-mNeon shows labeling of the microtubule root fibers associated with the paraflagellar region (arrow) and SPC. Scale bar: 2 μm.

Supplementary Figure 5 In-house generated αCP1 antibody labels the SPC. **(A)** Amino acid sequence of the chosen CP1 antigenic region (blue) with the N-terminal tag from the Pet32 LIC/EK vector (red). The black underlined region is the portion of the N-terminal tag that remains with the antigen after thrombin cleavage. **(B)** Purification of CP1 antigen for antibody generation. CP1 antigen (blue arrow) in the primary elution from Ni^2+^ column is thrombin digested, which cleaves off the N-terminal tag containing the 6x histidines (red arrow). The digested eluate is then passed through a Ni^2+^ column again, followed by a gentle elution with 10 mM imidazole. Pure, 6xHis tag-free CP1 antigen (green arrow) was eluted by this step and this purified antigen was then used for mouse inoculation. **(C)** Immunoblot of Parental and CP1-mNeon-Ty overexpressing mutant lysates showing the labeling of CP1-mNeon-Ty by polyclonal mouse αCP1 antibody. **(D)** SR-SIM IFA of Y strain epimastigotes showing αCP1 labeling of the SPC. Scale bars: 2 μm.

Supplementary Figure 6 Epimastigotes overexpressing CP3-mNeon exhibit a growth defect. **(A)** Growth assays of Parental (Y Strain), CP1-mNeon, CP2-mNeon, and CP3-mNeon epimastigotes. **(B)** Fold change in parasites during 48 h of exponential growth (24–72 h) shows a significant reduction in growth of the CP3-mNeon overexpressing mutants. ^*^*p* < 0.05.

Supplementary Table 1 Primers utilized in this work.
