# Supplementary figures and images for: Identification and Localization of the First Known Proteins of the Trypanosoma cruzi Cytostome Cytopharynx Endocytic Complex
Source: Front Cell Infect Microbiol. 2020 Jan 17;9:445. doi: 10.3389/fcimb.2019.00445 (PMC6978632; doi:10.3389/fcimb.2019.00445)

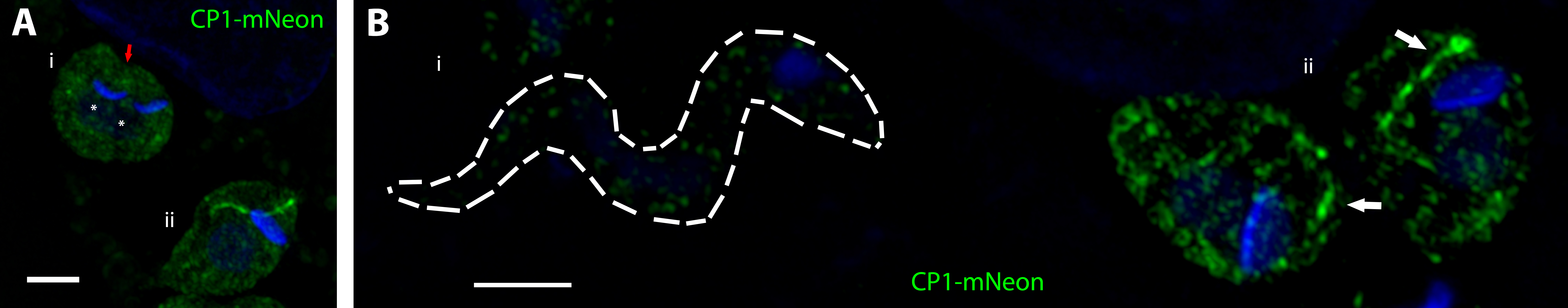

Supplement: Supplementary Figure 1 — SPC labeling is absent during cytokinesis and the trypomastigote stage. (A) Amastigote labeling of the SPC by CP1-mNeon (ii) is absent in amastigotes undergoing cytokinesis (i), determined by the presence of a cleavage furrow (red arrow) and two nuclei (asterisk). (B) The SPC labeling by CP1-mNeon seen in amastigotes (ii), is absent in a trypomastigote (i). Scale bars: 2 μm. [file Image_1.jpg]

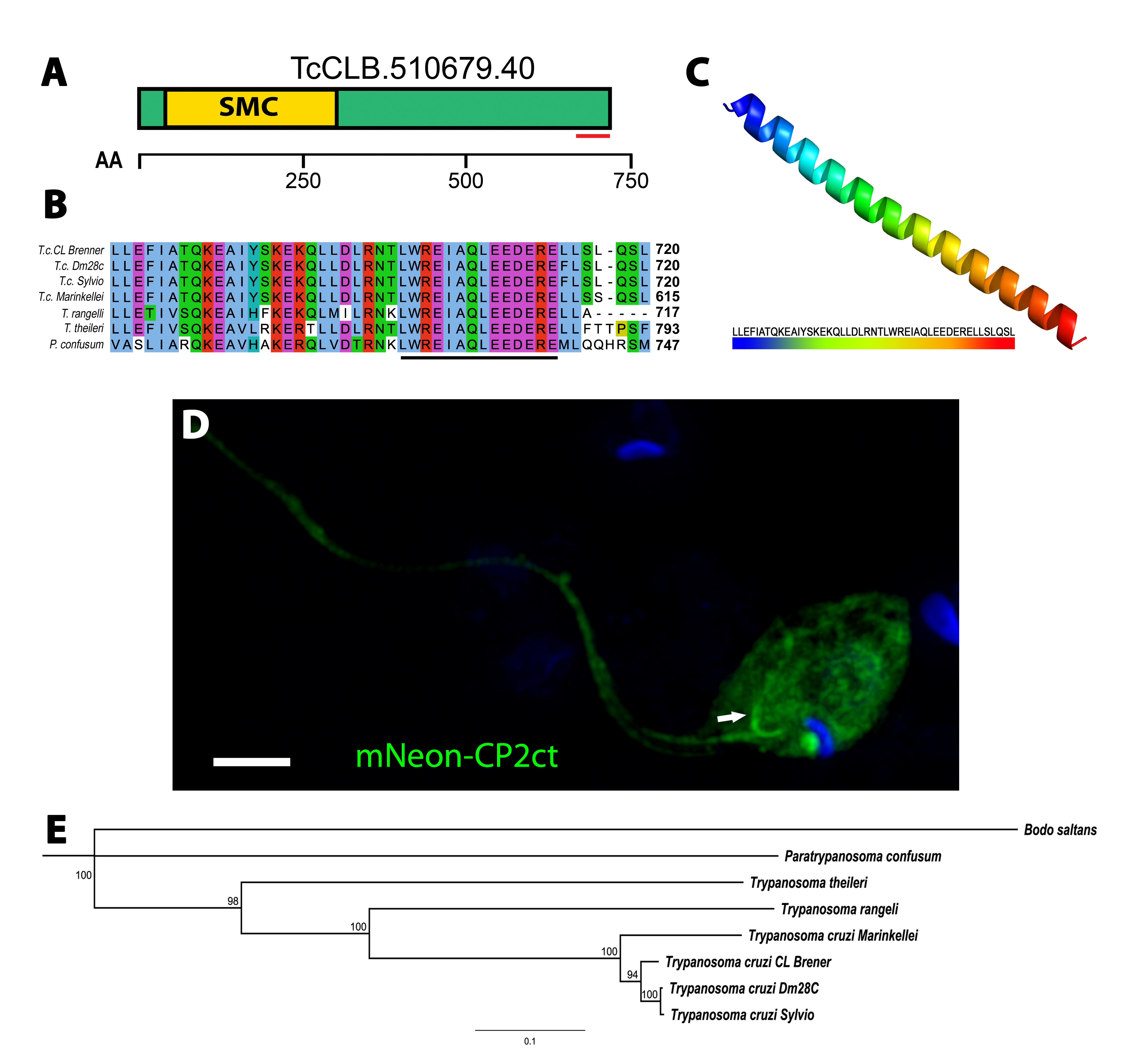

Supplement: Supplementary Figure 2 — The highly conserved C-terminal region of CP2 is sufficient to traffic mneon to the cytostome/cytopharynx complex. (A) Cartoon showing CP2 and its predicted SMC domain over an amino acid scale line. The red line annotates the highly conserved C-terminal region (675–720 aa). (B) Alignment of the highly conserved C-terminal region. The black line identifies a notable 14 amino acid region that maintains 100% identity among all orthologs, even in the more distantly related P. confusum. (C) The ITASSER predicted structure of the conserved C-terminal region is similar to that of a coil-coil protein interaction domain. The confidence score (range −5.0 lowest to +1.0 highest) of the predicted structure is high at −0.7. (D) Fluorescence image of an epimastigote overexpressing mNeon fused to the 45 aa CP2 C-terminal region (see B) shows SPC labeling (arrow). (E) A bootstrapped Jukes-Cantor neighbor-joining tree analysis of CP2 orthologs using the alignment from Supplementary Figure 3. The weakly related potential ortholog from B. saltans, which is missing the C-terminal region entirely was used as the outgroup. Branches are labeled with their bootstrap value. [file Image_2.JPEG]

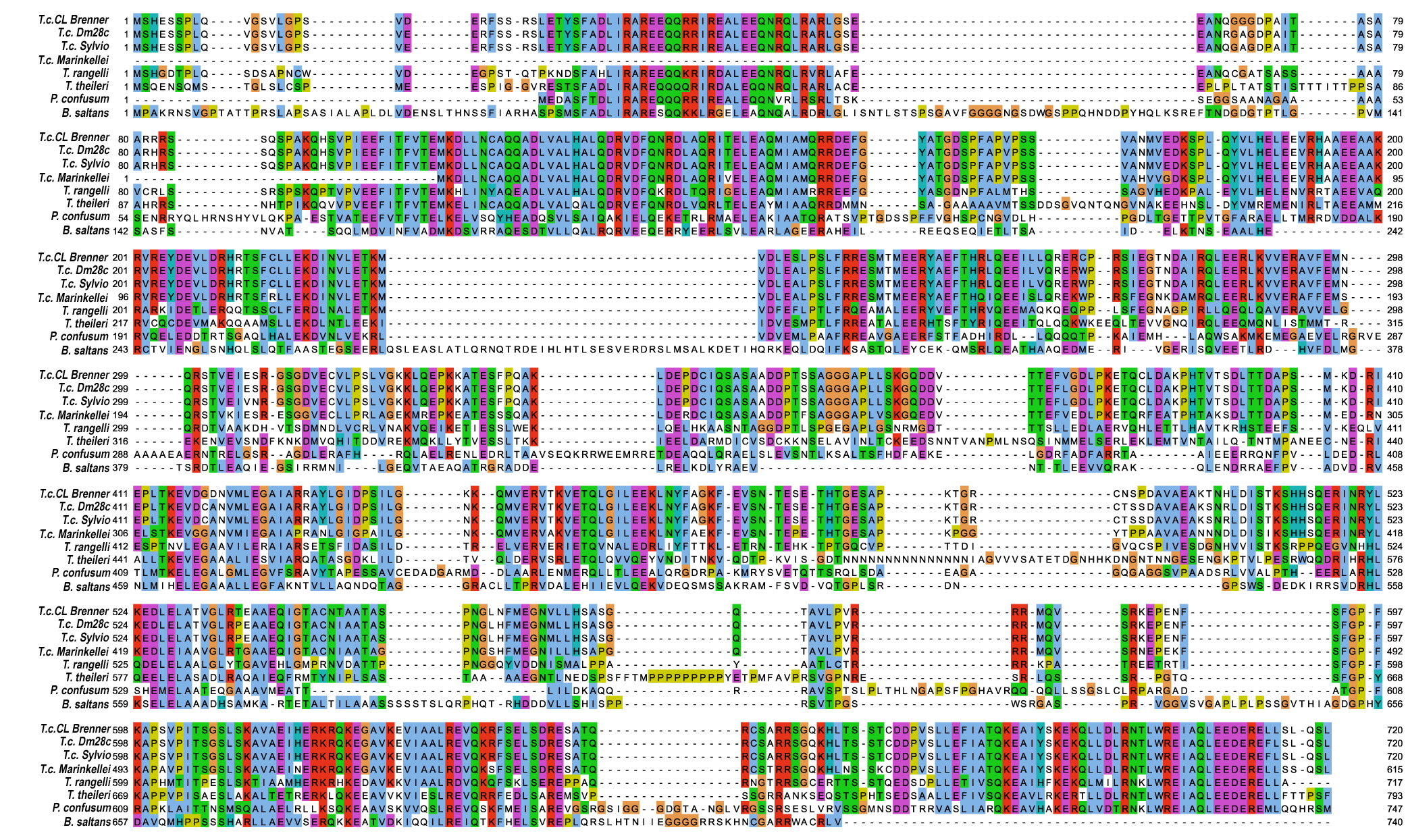

Supplement: Supplementary Figure 3 — Alignment of CP2 orthologs for tree analysis. T-coffee alignment of the CP2 orthologs from T. cruzi CL Brener (NCBI: XP_807919.1), T. cruzi Dm28c (Genbank: ESS69597.1), T.cruzi Sylvio (Genbank: EKG03371.1), T. cruzi Marinkellei (Genbank: EKF31948.1), T. rangelli (Genbank: ESL08911.1), T. Theileri (NCBI: XP_028885643.1), P. confusum (TriTrypDB: PCON_0003310), B. saltans (Genbank: CUG86305.1). [file Image_3.JPEG]

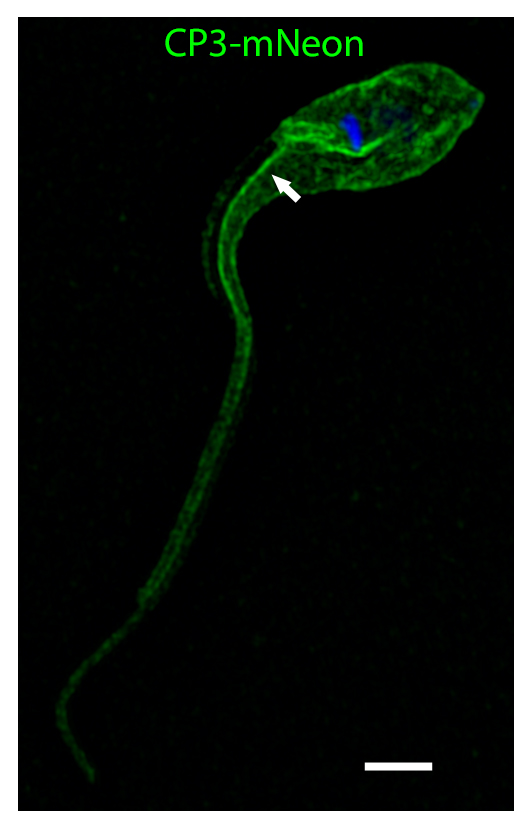

Supplement: Supplementary Figure 4 — CP3-mNeon labels structures reminiscent of the microtubule root fibers. Fluorescent image of the CP3-mNeon shows labeling of the microtubule root fibers associated with the paraflagellar region (arrow) and SPC. Scale bar: 2 μm. [file Image_4.JPEG]

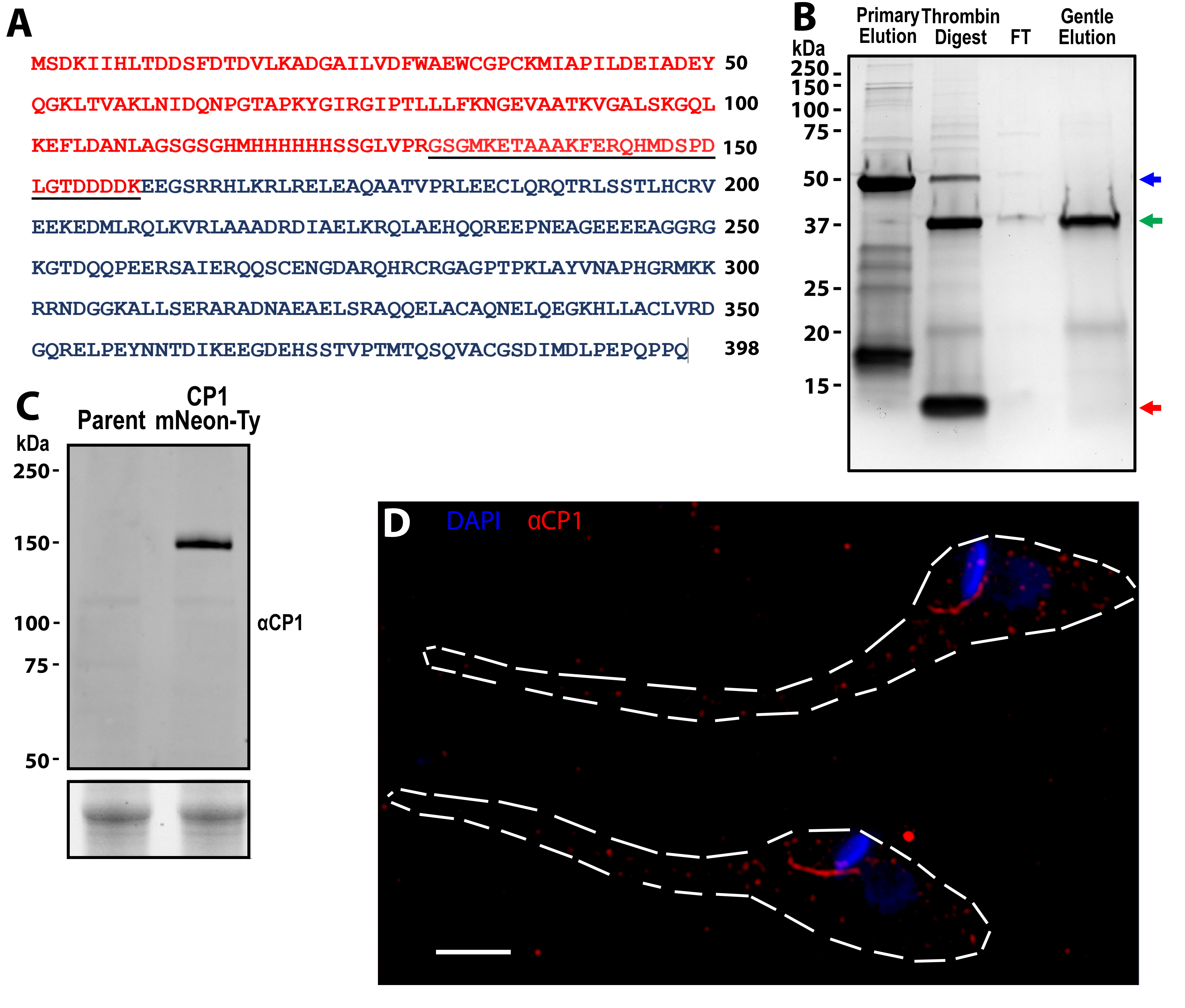

Supplement: Supplementary Figure 5 — In-house generated αCP1 antibody labels the SPC. (A) Amino acid sequence of the chosen CP1 antigenic region (blue) with the N-terminal tag from the Pet32 LIC/EK vector (red). The black underlined region is the portion of the N-terminal tag that remains with the antigen after thrombin cleavage. (B) Purification of CP1 antigen for antibody generation. CP1 antigen (blue arrow) in the primary elution from Ni2+ column is thrombin digested, which cleaves off the N-terminal tag containing the 6x histidines (red arrow). The digested eluate is then passed through a Ni2+ column again, followed by a gentle elution with 10 mM imidazole. Pure, 6xHis tag-free CP1 antigen (green arrow) was eluted by this step and this purified antigen was then used for mouse inoculation. (C) Immunoblot of Parental and CP1-mNeon-Ty overexpressing mutant lysates showing the labeling of CP1-mNeon-Ty by polyclonal mouse αCP1 antibody. (D) SR-SIM IFA of Y strain epimastigotes showing αCP1 labeling of the SPC. Scale bars: 2 μm. [file Image_5.jpg]

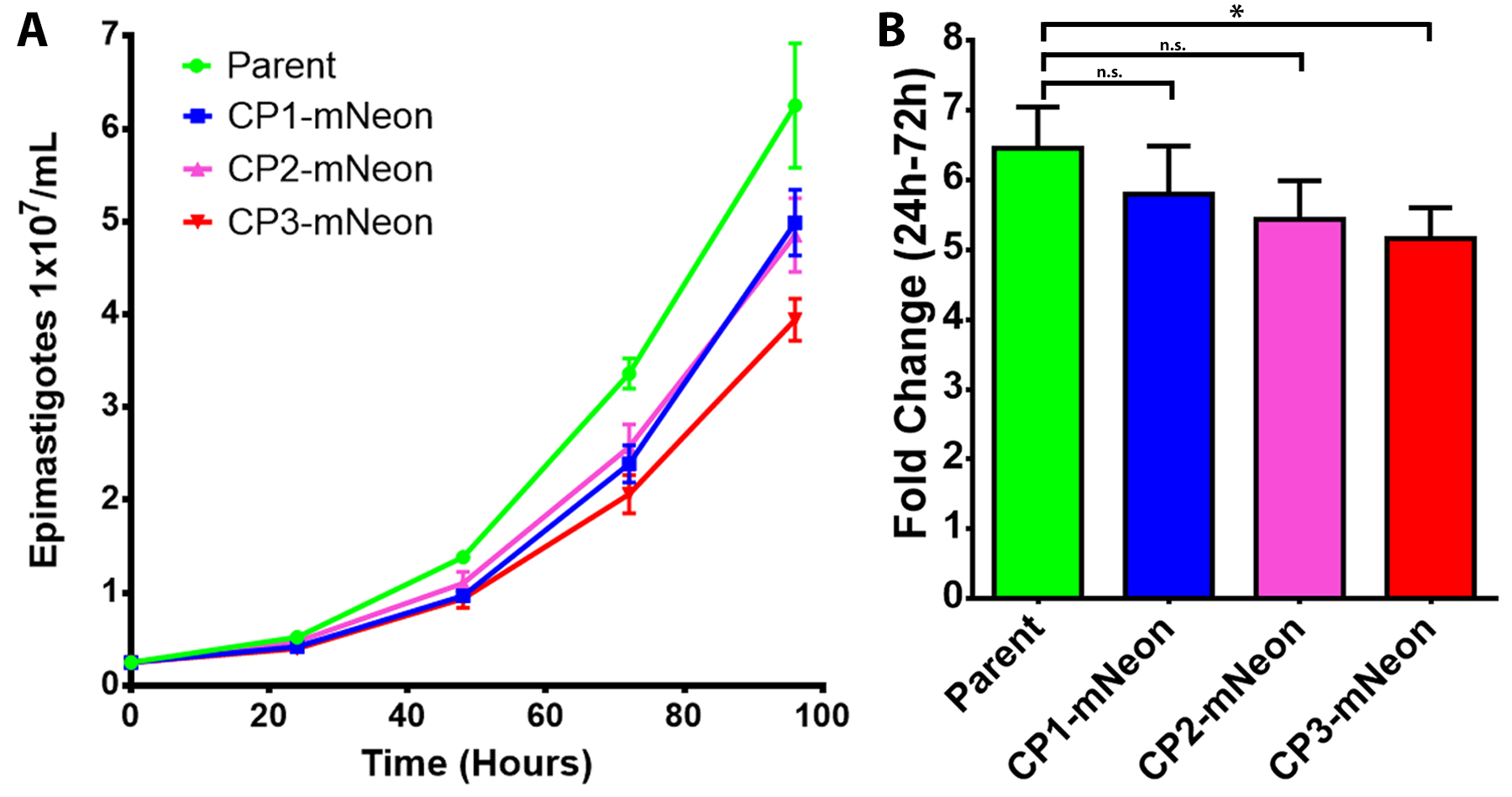

Supplement: Supplementary Figure 6 — Epimastigotes overexpressing CP3-mNeon exhibit a growth defect. (A) Growth assays of Parental (Y Strain), CP1-mNeon, CP2-mNeon, and CP3-mNeon epimastigotes. (B) Fold change in parasites during 48 h of exponential growth (24–72 h) shows a significant reduction in growth of the CP3-mNeon overexpressing mutants. *p < 0.05. [file Image_6.JPEG]
