# Supplementary material for: Identification and Localization of the First Known Proteins of the Trypanosoma cruzi Cytostome Cytopharynx Endocytic Complex
Source: Front Cell Infect Microbiol. 2020 Jan 17;9:445. doi: 10.3389/fcimb.2019.00445 (PMC6978632; doi:10.3389/fcimb.2019.00445)
Supplement: Supplementary Table 1 — Primers utilized in this work. [file Table_1.docx]

Supplementary Table 1:

| **Primer #** | **All Primers Oriented 5’ to 3’** |
| --- | --- |
| 1 | ggagatggaattagaagaggacgacaccgttgctccatgggaggtccacacgaaccaggacccgctcgattagtcgacaaagtgtgacaacgtcgcaccatgtgtagg |
| 2 | ggtgtataccgtgcttgtagagagaagtaatgacgctgggcctattcctttgccctcggacgagtgctggggcgtcggtttcc |
| 3 | gtgccacgactggaggaatgcctgcagcgg |
| 4 | gaaaacagaaggttcacatttcacatag |
| 5 | atcgagcgggtcctggttcgtgtggacctc |
| 6 | ccgttgctccatggggtgac |
| 7 | tataagttgtcttgtctagaatgcatgcatgtccgtgtctgtgcaatgcgtatctattta |
| 8 | attagaagaggacgacaccgttgctccatggggtgactggatggtgtccaaaggagaaga |
| 9 | gctctataagttgtcttgtctagaatgtcgcatgaaagcagtcc |
| 10 | cttcttctcctttggacactaaggattgtaacgataagagctcc |
| 11 | ctataagttgtcttgtctagaatgctgttttcatgcgtggagaagcagggcaagtaccgc |
| 12 | tcttcttctcctttggacacgtacttgccgccaataagc |
| 13 | cattctagacaagacaacttatagagc |
| 14 | gtgtccaaaggagaagaagac |
